# Medical Tuition by Correspondence

**Published:** 1910-10-29

**Authors:** 


### Medical Tuition by Correspondence.

The educational numbers which many medical journals—The Hospital included—publish annually about the beginning of September, and the prospectuses of the medical schools, contain no references to that old-established and flourishing institution, the medical "crammer." Nevertheless, private tuition of various kinds is a form of instruction to which a very considerable percentage of qualified medical men have at some time or other resorted while *in statu pupillari*. It is, naturally, in connection especially with examinations that this form of medical knowledge is assimilated; for just as no one can become a sound physician and surgeon simply by reading text-books or listening to lectures, but only by assiduous clinical study in the wards, so also can no "coach" instil more than the academic kind of knowledge which passes muster in examination halls.

At every medical school the "coach"—the word is used in no derogatory sense—abounds. Most frequently he is a young man, whose ambition is to become ultimately one of the visiting staff; or is a junior member of the latter, at whose door in Cavendish-land private patients have not yet begun to knock. In other words, he is a coach by accident, not by design; and his object in coaching is to tide over the lean years until he shall have his hands full of more lucrative work. Besides this type, there is the more permanent private teacher, who may be described as the professional crammer. In many cases these experts manage, or are members on the staff of, institutions where instruction is given for any and every form of degree and diploma; even such recondite sciences as public health are included within the syllabus, and advice regarding theses for M.D. is another specialty which some of them cultivate. A feature which distinguishes some of these highly organised and efficient teaching establishments is tuition by correspondence; and this art has by long practice been brought to a high pitch by many of those who have devoted themselves to it. Moreover, the fact that medical coaching by post has for long been successful is widely known, and is advertised in the announcements which may be seen in the advertisement columns of the medical press.

In such advertisements it is usual to specify numerical successes in recent examinations. It would appear that on occasions the statements contained in these lists of successes cannot be strictly relied on. This, at least, is the conclusion to be gathered from the perusal of a broadsheet recently circulated by the manager of one of the longest established and best known of these correspondence colleges. He alleges, in terms which are plainly meant to be libellous, that a rival institution is guilty of fraud in respect of its published lists of successes. As no suit for libel has yet followed, it may be supposed that the allegations in this circular contain at least a fair amount of truth. The specific act of fraud charged is in connection with the examination for the M.D. (Brux.) as granted to British practitioners. In May and June the complainant "coach" sent up five candidates for this degree; all passed, two of them with distinction. There were in all three more (successful) candidates, one of whom at least had no coaching. Yet the other institution claims during those two months to have passed twelve candidates, five with honours! The truth cannot be in both these assertions. Although it is dangerous to hear one side only, it seems as if the charge of fraud brought in the circular in question is well founded; and, therefore, it behoves any who are seeking this kind of medical teaching to be inquisitive regarding the claims of those who profess to give it.

